# Supplementary material for: Probing polaron-induced exciton quenching in TADF based organic light-emitting diodes
Source: Nat Commun. 2022 Jan 11;13:254. doi: 10.1038/s41467-021-27739-x (PMC8752634; doi:10.1038/s41467-021-27739-x)
Supplement: Supplementary file 1 — Supplementary Information [file 41467_2021_27739_MOESM1_ESM.pdf]

Supplementary Information

Probing polaron-induced exciton quenching in TADF based organic light-emitting diodes

Monirul Hasan,[1,2] Siddhartha Saggar,[1,2] Atul Shukla,[1,2] Fatima Bencheikh,[3] Jan Sobus,[1,2] Sarah K. M. McGregor,[2,4] Chihaya Adachi,[3,*] Shih-Chun Lo,[2,4,*] Ebinazar B. Namdas,[1,2,*]

[1]*School of Mathematics and Physics, The University of Queensland, Brisbane, QLD 4072, Australia*
[2]*Centre for Organic Photonics & Electronics, The University of Queensland, Brisbane, QLD 4072, Australia*
[3]*Center for Organic Photonics and Electronics Research, Kyushu University, Fukuoka 819-0395, Japan*
[4]*School of Chemistry and Molecular Biosciences, The University of Queensland, Brisbane, QLD 4072, Australia*

**Corresponding authors*

email: adachi@cstf.kyushu-u.ac.jp; s.lo@uq.edu.au; e.namdas@uq.edu.au

Contents

Supplementary Figures

Supplementary Fig. 1 | Chemical structure. a Thermally activated delayed fluorescent emitter 2,4,5,6-tetra(9*H*-carbazol-9-yl)isophthalonitrile (4CzIPN). b host 1,3-bis(*N*-carbazolyl)benzene (mCP)...... 4

Supplementary Fig. 2 | Hole-only diode (HOD) device structure with 4CzIPN. a HOD structure with mCP:5wt% 4CzIPN active layer. b Energy diagrams of the materials employed in the 4CzIPN HOD. 4

Supplementary Fig. 3 | Dual excitation setup. Schematic diagram of the dual excitation setup for the polaron- and field-induced quenching measurements. The sample was excited by CW He-Cd laser (excitation wavelength = 325 nm; excitation power = 50 μW). 5

Supplementary Fig. 4 | PL intensity reduction in 4CzIPN HODs. PL intensity as a function of applied voltages for 4CzIPN HODs. Under electrical bias no electroluminescence (EL) was observed, confirming unipolar charge transport in the devices.. 5

Supplementary Fig. 5 | Photophysical properties of mCP:4CzIPN blend. a Normalized absorption and PL spectra of mCP:4CzIPN blend films. b Excited state decay of the blend under optical excitation (excitation wavelength = 372 nm). The solid line represents the bi-exponential fit for the decay. Inset shows the instrument response function (IRF). 6

Supplementary Fig. 6 | Charge transport in mCP:4CzIPN HOD. a Experimental (circle) current density–voltage response and the fit with a numerical drift-diffusion model (solid line). b Corresponding voltage-dependent spatial polaron distribution in 4CzIPN based HOD device obtained from the drift-diffusion model under steady-state conditions, which provided the average polaron density at different applied voltages. 7

Supplementary Fig. 7 | Organic light-emitting diode (OLED) structure with 4CzIPN emitter, PL response from 4CzIPN OLEDs with increasing voltage and EL spectra. a Energy diagram of the 4CzIPN based OLED. b chemical structure of hole transport material poly(3,4-ethylenedioxythiophene):poly(styrenesulfonate) (PEDOT:PSS) and electron transport material 3,3',5,5'-tetra[(m-pyridyl)-phen-3-yl]biphenyl (BP4mPy). c PL intensity as a function of applied voltages under reverse bias for 4CzIPN OLED. Under reverse bias no EL was observed, confirming the absence of polaron-induced quenching in the device. d Steady-state EL spectra collected of the OLED at 250 cd m^{-2} under forward bias. 8

Supplementary Fig. 8 | Time-resolved PL response collected from 4CzIPN OLEDs. a PL response collected from OLEDs under reverse bias with pulsed optical excitation. The decrease in intensity under high voltage conditions (i.e., 15 V) is assumed to be due to the field-induced quenching. b Normalized PL intensity as a function of time. There is an increase of contribution from the delayed component, assumed to be due to less amount of quenching from triplet states. 9

Supplementary Fig. 9 | Non-doped 3-(9,9-dimethylacridin-10(9*H*)-yl)-9*H*-xanthen-9-one (ACRXTN) based HOD device characteristics. a HOD device structure with neat ACRXTN as the active layer. b Experimental (circle) and simulated (solid line) current density–voltage response for the HOD device, inset: Chemical structure of ACRXTN. c Voltage-dependent spatial polaron distribution obtain from neat ACRXTN HOD. 10

Supplementary Fig 10 | OLED structure and PL quenching in ACRXTN OLEDs. a OLED device structure with neat ACRXTN as the emissive layer. b Energy diagrams of the materials employed in

the ACRXTN OLED. c Chemical structure of electron transport material 2,2',2"-(1,3,5-benzinetriyl)-tris(1-phenyl-1-*H*-benzimidazole) (TPBi). d PL quenching yield as a function of the applied electric field. For the study of SPA and TPA rate in ACRXTN, field-induced quenching was not considered as the PL quenching yield due to electric field was negligible. 11

Supplementary Fig. 11 | PL quenching in ACRXTN HOD. a Energy diagram of the ACRXTN HOD. b The relative PL intensity under voltage-dependent constant optical excitation as a function of carrier density. Fits to the model reveal k_{SP} and k_{TP} as 1.3×10^{-12} cm^3 s^{-1} and 5.4×10^{-13} cm^3 s^{-1}, respectively. 12

Supplementary Fig. 12 | Device characteristics of ACRXTN OLEDs. a Current density−voltage−luminance plot. b EL spectra collected at 100 cd m^{-2}. c EQE−current density plot, the solid line represents the fit obtained from the OLED EQE roll-off model with k_{SP} and k_{TP} as 4×10^{-12} cm^3 s^{-1} and 6×10^{-13} cm^3 s^{-1}, respectively. 13

Supplementary Fig. 13 | Simulated singlet and triplet density versus current density plot. Solid and dash-dot lines represent with and without annihilation processes, respectively. 14

Supplementary Notes

Supplementary Note 1 | Calculation of TADF rate constants 15

Supplementary Note 2 | Calculation of average carrier density 15

Supplementary Note 3 | Onsager-Braun model 16

Supplementary Note 4 | Rubel model 17

Supplementary Note 5 | Calculation of exciton generation rate (I_x) 17

Supplementary Tables

Supplementary Table 1 | Summary of rate constants and PL efficiencies for mCP:4CzIPN blend films. 18

Supplementary Table 2 | Summary of the parameters used for the simulation of 4CzIPN HOD device. 18

Supplementary Table 3 | Summary of the fitting parameters for the Rubel model for mCP:4CzIPN.19

Supplementary Table 4 | EL properties of 4CzIPN OLEDs. 19

Supplementary Table 5 | Summary of the parameters used for the current density−voltage simulation of ACRXTN HOD device. 20

Supplementary Table 6 | EL properties of ACRXTN OLEDs. 20

Supplementary Table 7 | Summary of parameters used to fit EQE−current density fit for neat ACRXTN OLED. 21

Supplementary References 22

Supplementary Figures

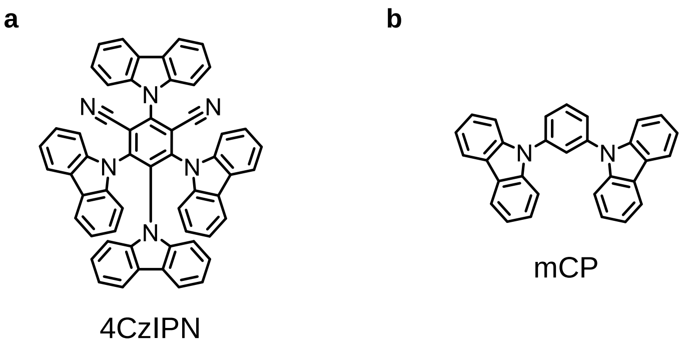

Supplementary Fig. 1 | Chemical structure. a Thermally activated delayed fluorescent emitter 2,4,5,6-tetra(9*H*-carbazol-9-yl)isophthalonitrile (4CzIPN). **b** host 1,3-bis(*N*-carbazolyl)benzene (mCP).

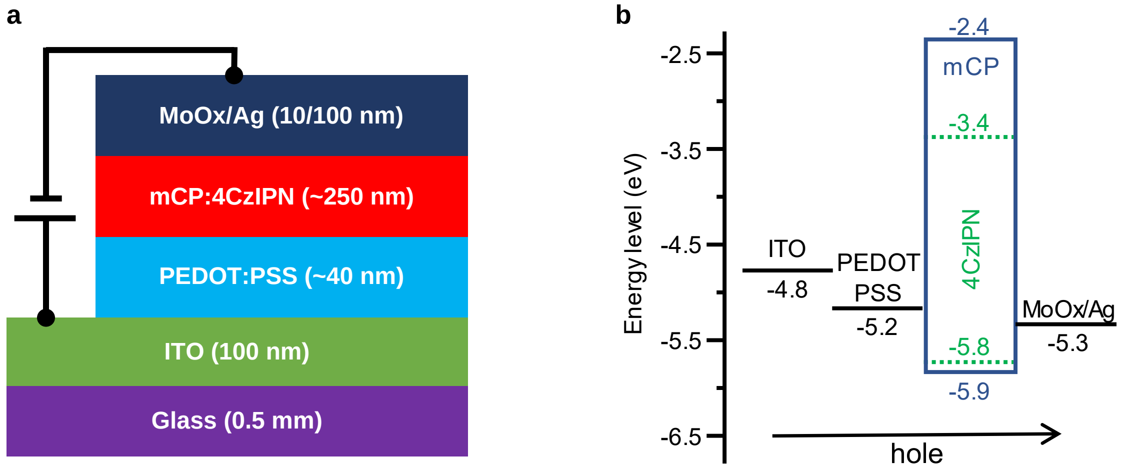

Supplementary Fig. 2 | Hole-only diode (HOD) device structure with 4CzIPN. a HOD structure with mCP:5wt% 4CzIPN active layer. **b** Energy diagrams of the materials employed in the 4CzIPN HOD.

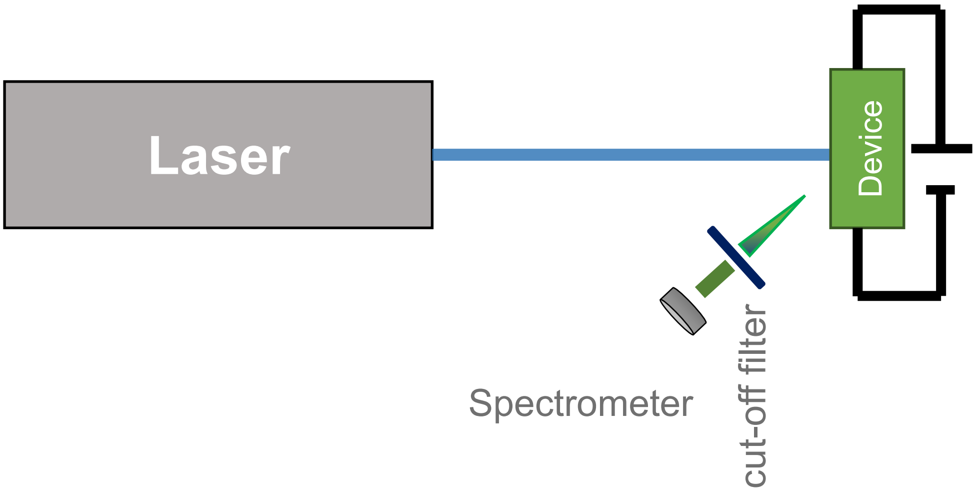

Supplementary Fig. 3 | Dual excitation setup. Schematic diagram of the dual excitation setup for the polaron- and field-induced quenching measurements. The sample was excited by CW He-Cd laser (excitation wavelength = 325 nm; excitation power = 50 μW).

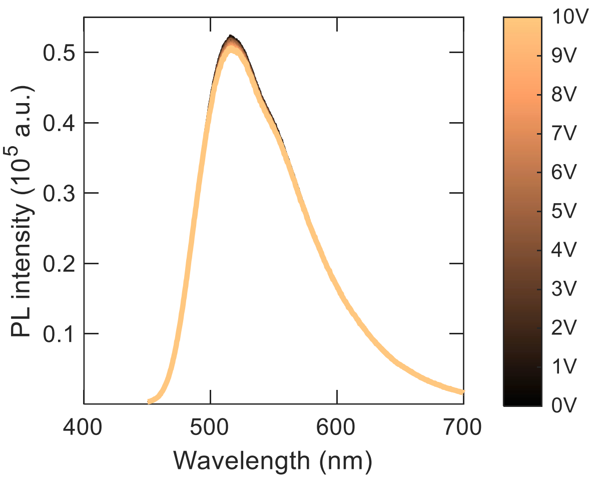

Supplementary Fig. 4 | PL intensity reduction in 4CzIPN HODs. PL intensity as a function of applied voltages for 4CzIPN HODs. Under electrical bias no electroluminescence (EL) was observed, confirming unipolar charge transport in the devices.

a b

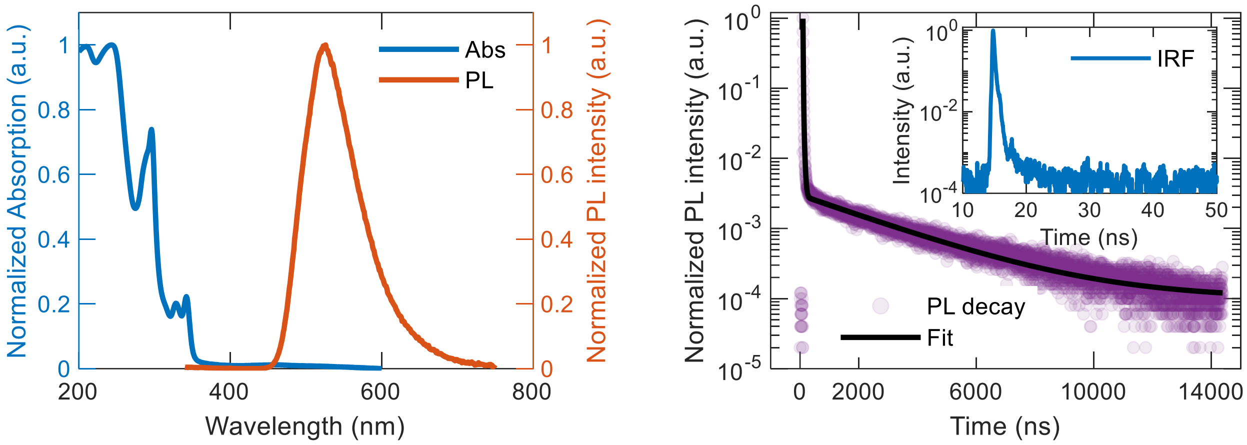

Supplementary Fig. 5 | Photophysical properties of mCP:4CzIPN blend. a Normalized absorption and PL spectra of mCP:4CzIPN blend films. **b** Excited state decay of the blend under optical excitation (excitation wavelength = 372 nm). The solid line represents the bi-exponential fit for the decay. Inset shows the instrument response function (IRF).

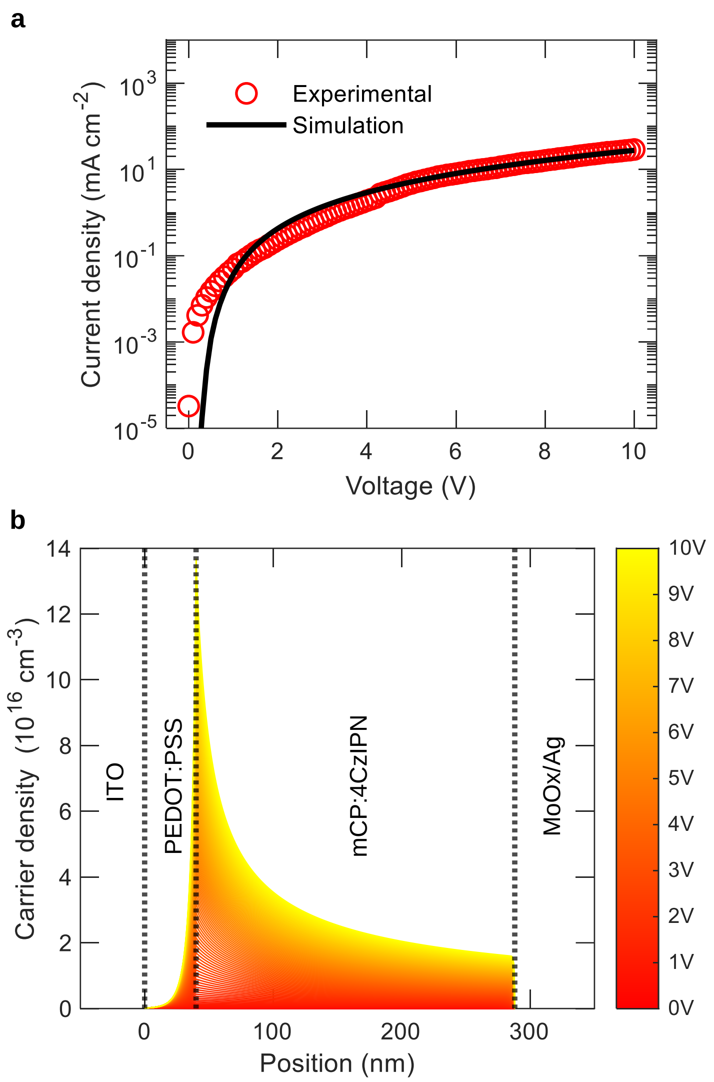

Supplementary Fig. 6 | Charge transport in mCP:4CzIPN HOD. a Experimental (circle) current density−voltage response and the fit with a numerical drift-diffusion model (solid line). **b** Corresponding voltage-dependent spatial polaron distribution in 4CzIPN based HOD device obtained from the drift-diffusion model under steady-state conditions, which provided the average polaron density at different applied voltages.

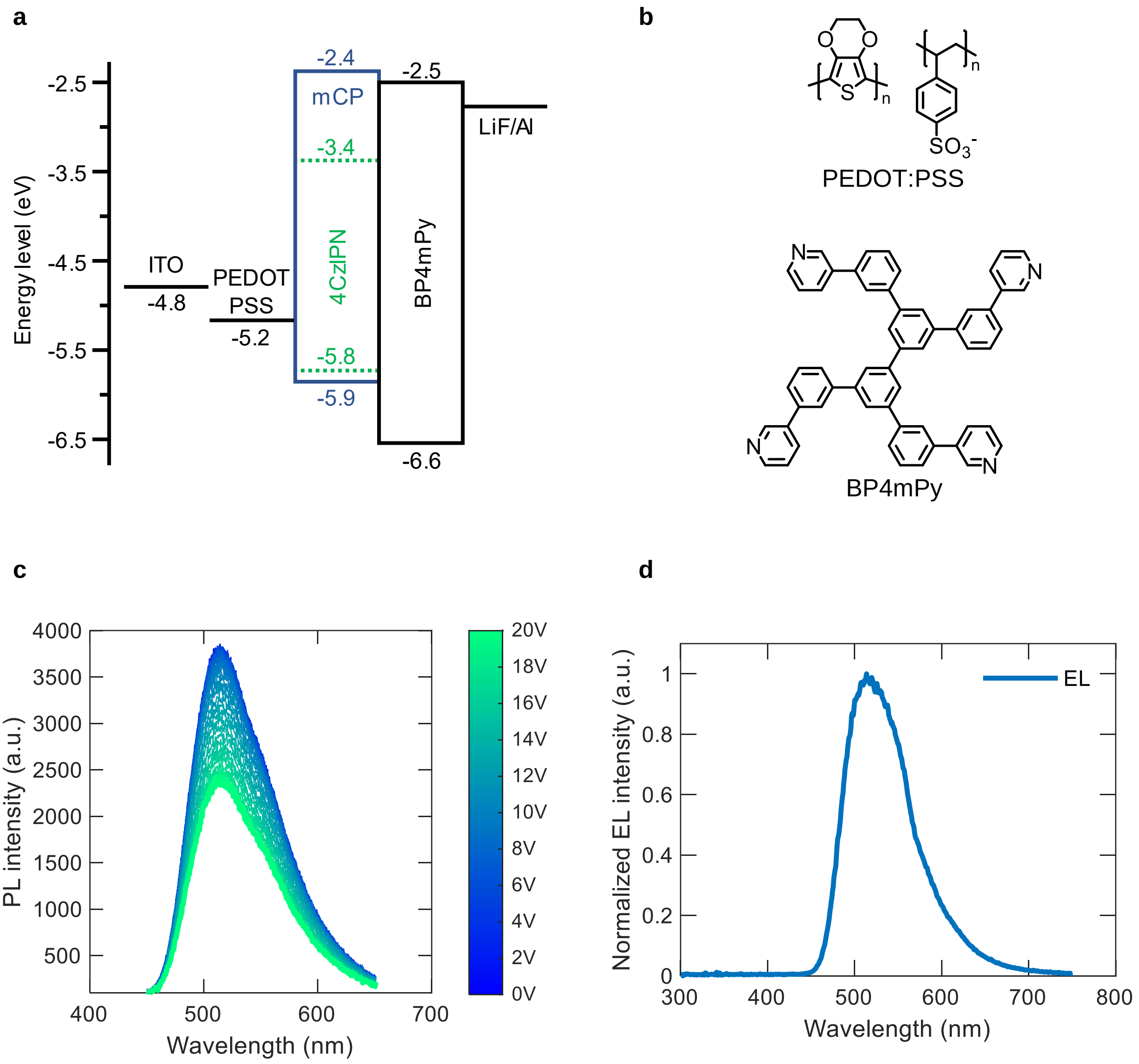

Supplementary Fig. 7 | Organic light-emitting diode (OLED) structure with 4CzIPN emitter, PL response from 4CzIPN OLEDs with increasing voltage and EL spectra. a Energy diagram of the 4CzIPN based OLED. **b** chemical structure of hole transport material poly(3,4-ethylenedioxythiophene):poly(styrenesulfonate) (PEDOT:PSS) and electron transport material 3,3',5,5'-tetra[(m-pyridyl)-phen-3-yl]biphenyl (BP4mPy). **c** PL intensity as a function of applied voltages under reverse bias for 4CzIPN OLED. Under reverse bias no EL was observed, confirming the absence of polaron-induced quenching in the device. **d** Steady-state EL spectra collected of the OLED at 250 cd m^{-2} under forward bias.

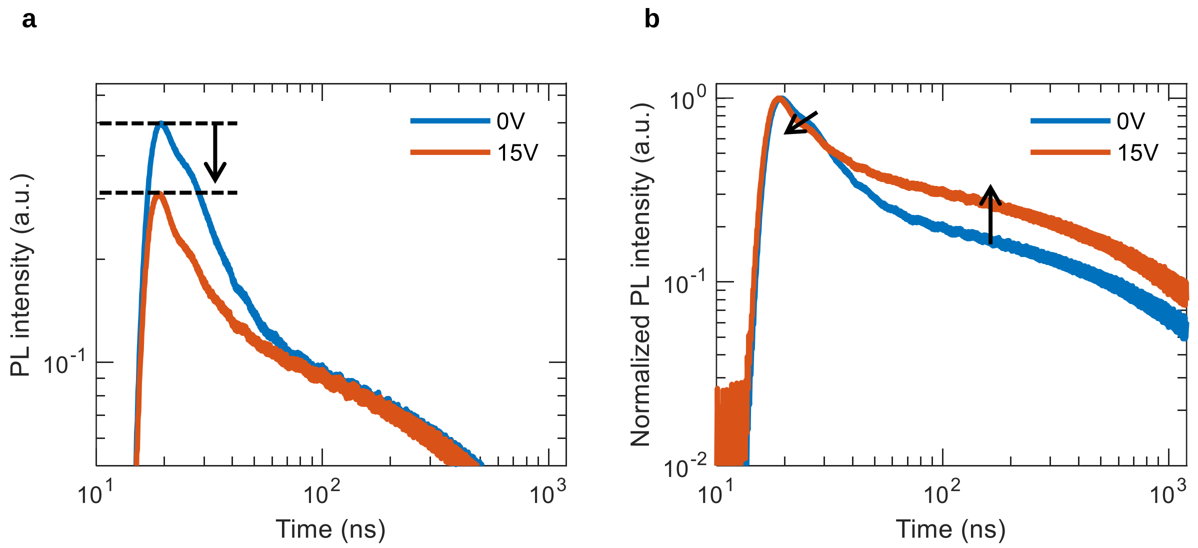

Supplementary Fig. 8 | Time-resolved PL response collected from 4CzIPN OLEDs. **a** PL response collected from OLEDs under reverse bias with pulsed optical excitation. The decrease in intensity under high voltage conditions (i.e., 15 V) is assumed to be due to the field-induced quenching. **b** Normalized PL intensity as a function of time. There is an increase of contribution from the delayed component, assumed to be due to less amount of quenching from triplet states.

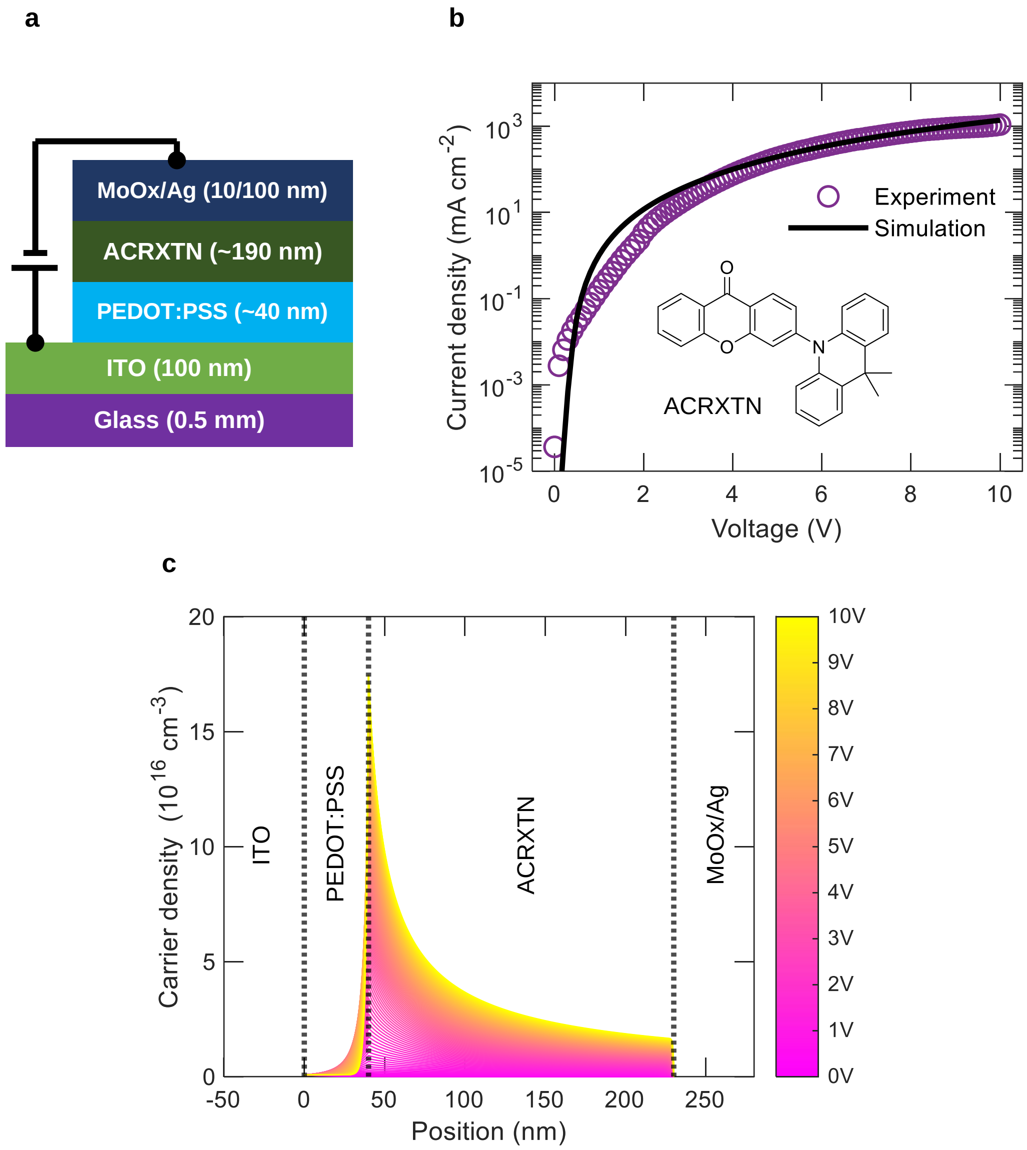

Supplementary Fig. 9 | Non-doped 3-(9,9-dimethylacridin-10(9*H*)-yl)-9*H*-xanthen-9-one (ACRXTN) based HOD device characteristics. a HOD device structure with neat ACRXTN as the active layer. **b** Experimental (circle) and simulated (solid line) current density–voltage response for the HOD device, inset: Chemical structure of ACRXTN. **c** Voltage-dependent spatial polaron distribution obtain from neat ACRXTN HOD.

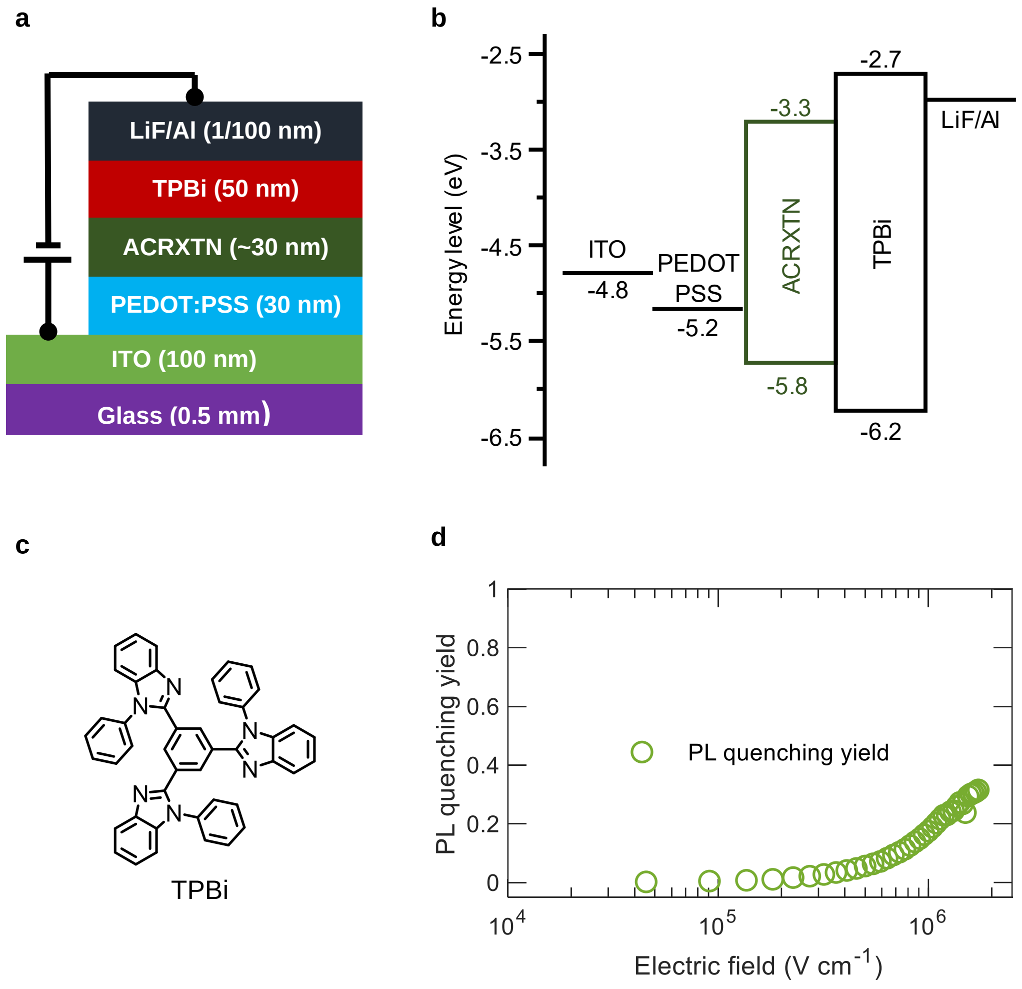

Supplementary Fig 10 | OLED structure and PL quenching in ACRXTN OLEDs. **a** OLED device structure with neat ACRXTN as the emissive layer. **b** Energy diagrams of the materials employed in the ACRXTN OLED. **c** Chemical structure of electron transport material 2,2',2"-(1,3,5-benzinetriyl)-tris(1-phenyl-1-*H*-benzimidazole) (TPBi). **d** PL quenching yield as a function of the applied electric field. For the study of SPA and TPA rate in ACRXTN, field-induced quenching was not considered as the PL quenching yield due to electric field was negligible.

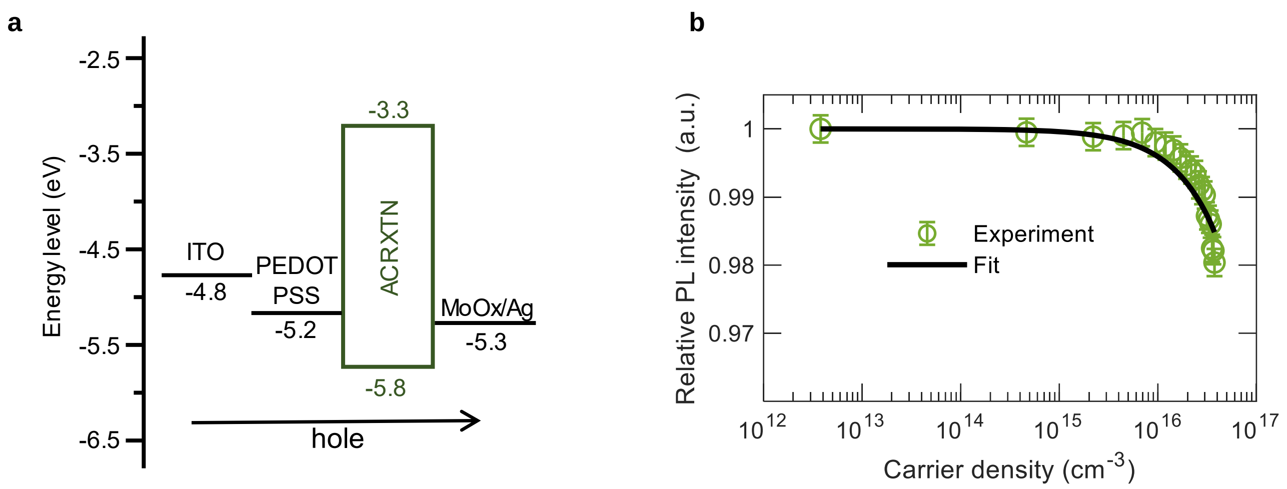

Supplementary Fig. 11 | PL quenching in ACRXTN HOD. a Energy diagram of the ACRXTN HOD. **b** The relative PL intensity under voltage-dependent constant optical excitation as a function of carrier density. Fits to the model reveal k_{SP} and k_{TP} as 1.3×10^{-12} cm^3 s^{-1} and 5.4×10^{-13} cm^3 s^{-1}, respectively.

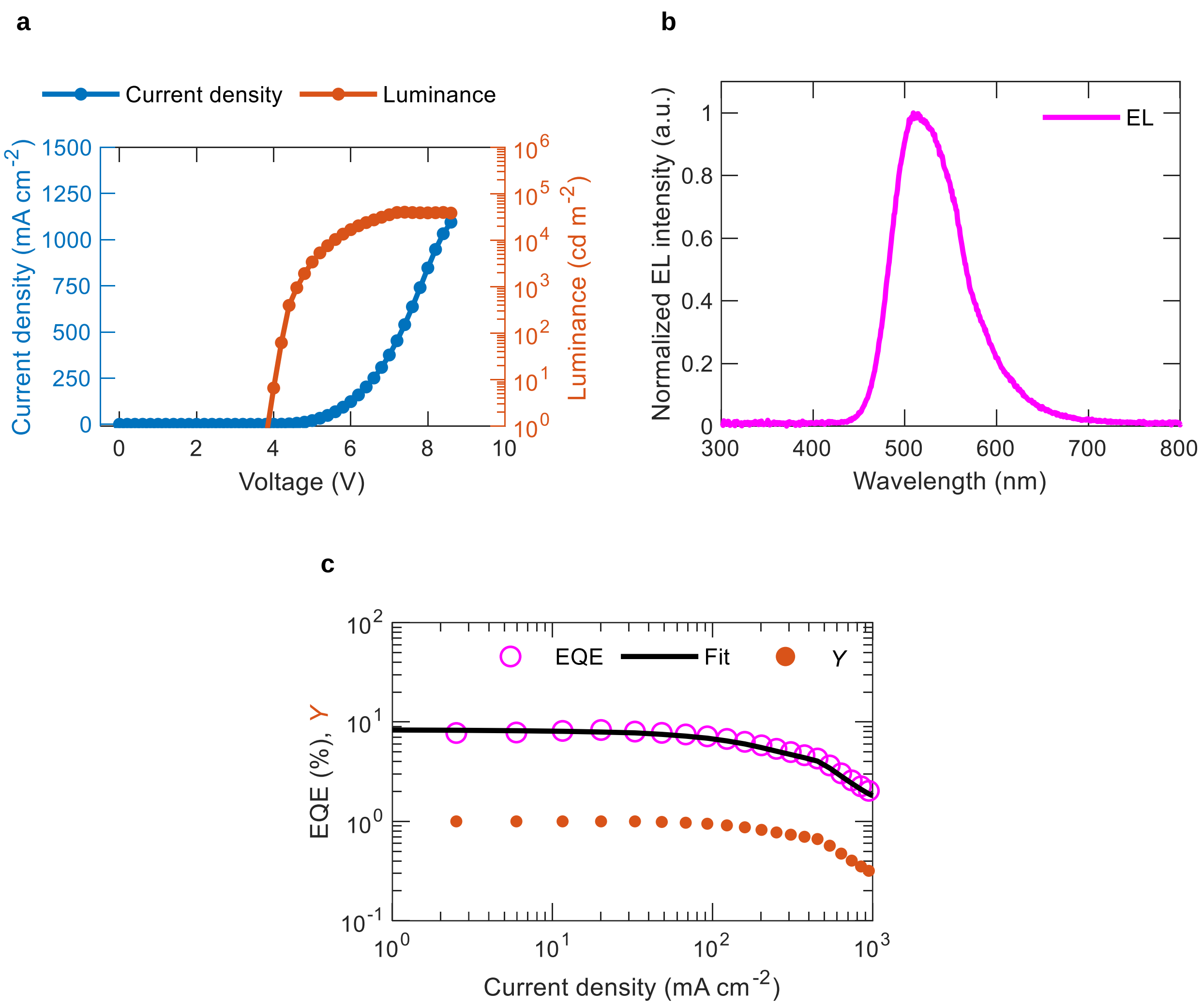

Supplementary Fig. 12 | Device characteristics of ACRXTN OLEDs. a Current density−voltage−luminance plot. **b** EL spectra collected at 100 cd m^{-2}. **c** EQE−current density plot, the solid line represents the fit obtained from the OLED EQE roll-off model with k_{SP} and k_{TP} as 4 × 10^{-12} cm^3 s^{-1} and 6 × 10^{-13} cm^3 s^{-1}, respectively.

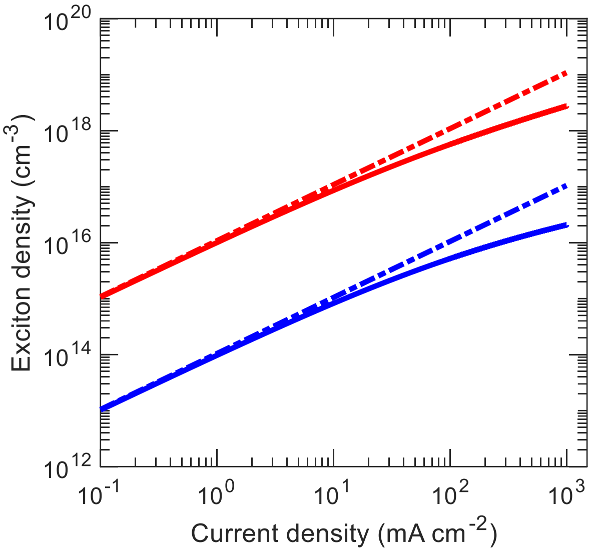

Supplementary Fig. 13 | Simulated singlet and triplet density versus current density plot. Solid and dash-dot lines represent with and without annihilation processes, respectively.

Supplementary Notes

Supplementary Note 1 | Calculation of TADF rate constants

From photophysical data, we calculated the rate constants for mCP:4CzIPN under optical pumping using the Eqs. (S1)–(S4), given as[1]

$$k_S = k_P \Phi_P, \quad \text{(S1)}$$

$$k_{ISC} = k_P(1 - \Phi_P), \quad \text{(S2)}$$

$$k_{RISC} = \frac{k_D k_P}{k_{ISC}} \frac{\Phi_D}{\Phi_P}, \quad \text{(S3)}$$

$$k_T = k_D - \Phi_P k_{RISC}, \quad \text{(S4)}$$

where k_S, k_{ISC}, k_{RISC}, and k_T are the singlet decay rate constant from the singlet state to the ground state, intersystem crossing (ISC) rate constant from the singlet state to the triplet state, reverse intersystem crossing (RISC) rate constant from the triplet state to the singlet state, and non-radiative triplet decay rate constant from the triplet state to the ground state, respectively; k_P and k_D are the prompt and delayed rate constant, respectively; Φ_P and Φ_D are the quantum efficiencies of the prompt and delayed components, respectively. Supplementary Table 1 summarises all the density-independent rate constants and PL efficiencies of the mCP:4CzIPN blend.

Supplementary Note 2 | Calculation of average carrier density

To extract the zero-field mobility and the spatial carrier distribution in the single-carrier devices, a one-dimensional (1D) drift-diffusion approach was used[2, 3]. A description of the model is given below as

$$\nabla^2 V(x) = -\frac{q}{\varepsilon_0 \varepsilon_r}[p(x)], \quad \text{(S5)}$$

$$\frac{\partial p(x)}{\partial t} = \frac{1}{q}\frac{\partial J_p(x)}{\partial x}, \quad \text{(S6)}$$

$$J_p(x) = qp(x)\mu_h f(x) + q\mu_h V_t \frac{\partial p(x)}{\partial x}, \quad \text{(S7)}$$

$$f(x) = -\frac{\partial V(x)}{\partial x}, \quad \text{(S8)}$$

$$V = V_{ext} - V_{bi} = \int_0^L f(x)dx, \quad \text{(S9)}$$

$$\mu_h(f) = \mu_{h0} \exp\left(\sqrt{\frac{f}{f_0}}\right), \quad \text{(S10)}$$

where V is electric potential as a function of position x; ε_0 is the permittivity of free space and ε_r the relative permittivity; p, q, J_p, and f is the hole density, elementary charge, hole current density, and external electric field, respectively; $V_t = k_B T/q$ is the thermal voltage, where k_B is the Boltzmann constant and T is the temperature; V_{ext} and V_{bi} are the external applied bias and built-in voltage, respectively; μ_h, μ_{h0}, and f_0 is the field-dependent mobility, zero-field mobility, and characteristic field, respectively. For the simulation, the total thickness of the organic layers was divided into 0.25 nm grid spacing with a simulation volume of 1151 points from the anode located at $x = 0$ and cathode at $x = L$. Using Gummel iteration scheme Eqs. (S5)–(S10), was solved self-consistently. The complete set of the parameter used for the simulation to obtain the best $J - V$ fit is given below in Supplementary Table 2 and Table 5 for mCP:4CzIPN and non-doped ACRXTN HODs, respectively. Spatial charge distributions obtained under each applied voltage were averaged to obtain average polaron density in the emissive layer.

Supplementary Note 3 | Onsager-Braun model

From the Onsager-Braun model, the electron-hole pair dissociation rate under an applied external electric field (f) can be assumed as[4]

$$R(f) = \frac{3\gamma}{4\pi r^3} \exp\left(-\frac{E_b}{k_B T}\right) \frac{J_\alpha\left(2\sqrt{-2b}\right)}{\sqrt{-2b}}, \quad \text{(S11)}$$

$$\gamma = \frac{q(\mu_h + \mu_e)}{\varepsilon_0 \varepsilon_r}, \quad \text{(S12)}$$

$$E_b = \frac{q^2}{4\pi\varepsilon_0\varepsilon_r r}, \quad \text{(S13)}$$

$$b = \frac{q^3 f}{8\pi\varepsilon_0\varepsilon_r (k_B T)}, \quad \text{(S14)}$$

where γ is the Langevin recombination rate, r is the electron-hole separation, E_b exciton binding energy, k_B is the Boltzmann constant, T is the temperature, J_α is the Bessel function of order one, μ_e is the electron mobility, and all the other terms hold their previous descriptions. The dissociation probability of electron-hole pair can be written as

$$P(f) = \frac{R(f)}{R(f) + \tau^{-1}}, \quad \text{(S15)}$$

where τ is the average exciton lifetime. These equations can be used to calculate exciton binding energy and field-induced quenching rate in TADF.

Supplementary Note 4 | Rubel model

In the case of the Rubel model, we have calculated electric field dependent electron-hole pair dissociation rate $R(f)$ as[5]

$$R(f) = \left[\sum_{j=1}^{n-1} \frac{1}{a_j(f)} \exp\left(\frac{E_j(f) - E_1}{k_B T/q}\right)\right]^{-1}, \tag{S16}$$

where, E_1 and E_j are the energies at the first and j-th sites in the Coulomb potential, respectively. The exciton binding energy can be calculated from E_1 in the absence of disorder and electric field. The term a_j is the Miller–Abrahams transition hopping rate from site j to site $j+1$, and is given by

$$a_j(f) = \nu_0 exp\left(-\frac{2(r_{j+1} - r_j)}{\alpha} - \frac{E_{j+1} - E_j + |E_{j+1} - E_j|}{2k_B T/q}\right), \tag{S17}$$

where, ν_0 is the attempt-to-escape frequency and α is the localization length. The first term in the argument of the exponential function is associated with the tunneling between sites that are spatially separated by $r_{j+1} - r_j$. The spatial disorder is included by adding a random number x_j chosen from a range between $\pm\delta r$ to the equidistant spacing r_0 between sites in such a way that $r_j = jr_0 + x_j$. The second term assumes that the jumps to higher energy are thermally activated and downward hops occur with a probability equal to one. Here, E_j can be given as

$$E_j(f) = E_j^0(\sigma) - \frac{q^2}{4\pi\varepsilon_r\varepsilon_0 r_j} - qfr_j. \tag{S18}$$

The effects of the energetic disorder are taken into account through E_j^0, which is a random value chosen from a Gaussian distribution centered at zero with a standard deviation σ. The simulation was run 10^4 times to average the calculated dissociation rate coefficient. The dissociation probability was calculated with Eq. (S15) using $R(f)$ and average lifetime τ. All the fitting parameters for this model are summarized in Supplementary Table 3.

Supplementary Note 5 | Calculation of exciton generation rate (I_X)

The generated singlet population under optical excitation was calculated as

$$n = \frac{E}{V} \frac{\lambda}{hc} (1 - 10^{-A}), \tag{S19}$$

where E is the energy of the laser beam, V is the volume of the film, λ is the excitation wavelength, A is the absorbance of the film, c is the speed of light and h is Planck's constant. For convenience, we assumed a uniform density of excitons throughout the beam profile. The volume of the film was calculated from device pixel area and active layer thickness.

Supplementary Tables

Supplementary Table 1 | Summary of rate constants and PL efficiencies for mCP:4CzIPN blend films.

k_S [s^{-1}]×10^7	k_{ISC} [s^{-1}]×10^7	k_{RISC} [s^{-1}]×10^5	k_T [s^{-1}]×10^4	QY [%]	ϕ_P [%]	ϕ_D [%]	ϕ_{ISC} [%]	ϕ_{RISC} [%]
3.0	4.14	5.95	9.50	84	41.94	42.05	58.05	72.44

Supplementary Table 2 | Summary of the parameters used for the simulation of 4CzIPN HOD device.

Parameter	Value	Source
Density of states (DOS)	10^{21} cm^{-3}	Fit
Anode (ITO) work function	− 4.8 eV	Ref.[6]
Cathode (MoOx/Ag) work function	− 5.3 eV	Ref.[7]
PEDOT:PSS HOMO level	− 5.2 eV	Ref.[8]
Carrier mobility PEDOT:PSS	4.5×10^{-2} $cm^2\,V^{-1}\,s^{-1}$	Ref.[9]
Relative permittivity	3	Ref.[10]
mCP HOMO level	− 5.9 eV	Ref.[11]
Active layer mobility (mCP:4CzIPN)	1×10^{-5} $cm^2\,V^{-1}\,s^{-1}$	Fit
Characteristics field	1.13×10^6 $V\,cm^{-1}$	Fit

Supplementary Table 3 | Summary of the fitting parameters for the Rubel model for mCP:4CzIPN.

Parameter name	Symbol	value
Relative permittivity	ε	3
Attempt-to-escape frequency	ν_0	10^{13} s^{-1}
Site separation	r_0	0.75 nm
Localization length	α_l	0.8 nm
Spatial disorder	δr	0.1 nm
Energetic disorder	σ	0.16 meV
Binding energy	E_b	0.48 meV

Supplementary Table 4 | EL properties of 4CzIPN OLEDs.

$\lambda_{max}{}^{a}$ [nm]	$V_{on}{}^{b}$ [V]	$L_{max}{}^{c}$ [cd m^{-2}]	$EQE_{max}{}^{d}$ [%]	$EQE_{5000}{}^{e}$ [%]	$CE_{max}{}^{f}$ [cd A^{-1}]	CIE^{g} [x,y]
513	4.5	~1.4×10^{4}	18.9	13.4	31.40	0.29, 0.57

[a]EL emission maximum. [b] Device turn-on voltage. [c] Maximum luminance. [d] Maximum EQE. [e]EQE at 5000 cd m^{-2}. [f] Maximum current efficiency. [g] Commission Internationale de l'Eclairage (CIE) color chromaticity coordinates measured at 100 cd m^{-2}.

Supplementary Table 5 | Summary of the parameters used for the current density−voltage simulation of ACRXTN HOD device.

Parameter	Value	Source
Density of states (DOS)	5×10^{21} cm^{-3}	Fit
Anode (ITO) work function	− 4.8 eV	Ref.[6]
Cathode (MoOx/Ag) work function	− 5.3 eV	Ref.[7]
PEDOT:PSS HOMO level	− 5.2 eV	Ref.[8]
Carrier mobility PEDOT:PSS	4.5×10^{-2} cm^2 V^{-1} s^{-1}	Ref.[9]
Relative permittivity	3	Ref.[10]
ACRXTN HOMO level	− 5.8 eV	Ref.[12]
Active layer mobility (ACRXTN)	5.4×10^{-5} cm^2 V^{-1} s^{-1}	Fit
Characteristics field (ACRXTN)	8.6×10^{4} V cm^{-1}	Fit

Supplementary Table 6 | EL properties of ACRXTN OLEDs.

$\lambda_{max}{}^{a}$ [nm]	$V_{on}{}^{b}$ [V]	$L_{max}{}^{c}$ [cd m^{-2}]	$EQE_{max}{}^{d}$ [%]	$EQE_{5000}{}^{e}$ [%]	$CE_{max}{}^{f}$ [cd A^{-1}]	CIE^{g} [x,y]
509	4	~4×10^{4}	8.3	8.01	16.9	0.27, 0.56

[a]EL emission maximum. [b] Device turn-on voltage. [c] Maximum luminance. [d] Maximum EQE. [e]EQE at 5000 cd m^{-2}. [f] Maximum current efficiency. [g] Commission Internationale de l'Eclairage (CIE) color chromaticity coordinates measured at 100 cd m^{-2}.

Supplementary Table 7 | Summary of parameters used to fit EQE−current density fit for neat ACRXTN OLED.

Parameter	Value	Source
k_S	8.8×10^{6} s^{-1}	Ref.[13]
k_{ISC}	1.7×10^{7} s^{-1}	Ref.[13]
k_{RISC}	1.9×10^{6} s^{-1}	Ref.[13]
k_T	3.4×10^{5} s^{-1}	Ref.[13]
Singlet-singlet annihilation rate (k_{SS})	2.4×10^{-12} cm^{3} s^{-1}	Ref.[13]
Singlet-triplet annihilation rate (k_{ST})	1.2×10^{-12} cm^{3} s^{-1}	Ref.[13]
Triplet-triplet annihilation rate (k_{TT})	9.7×10^{-14} cm^{3} s^{-1}	Ref.[13]
Electron mobility (μ_e)	1×10^{-5} cm^{2} V^{-1} s^{-1}	Ref.[13]
Hole mobility (μ_h)	5.4×10^{-5} cm^{2} V^{-1} s^{-1}	Fit
Recombination zone width (d)	15 nm	Fit
Singlet-polaron annihilation rate (k_{Sp})	4×10^{-12} cm^{3} s^{-1}	Fit
Triplet-polaron annihilation rate (k_{TP})	6×10^{-13} cm^{3} s^{-1}	Fit

Supplementary References

1. Goushi K., Yoshida K., Sato K., & Adachi C. Organic light-emitting diodes employing efficient reverse intersystem crossing for triplet-to-singlet state conversion. *Nat. Photonics* **6**, 253 (2012).

2. Knapp E., Häusermann R., Schwarzenbach H. U., & Ruhstaller B. Numerical simulation of charge transport in disordered organic semiconductor devices. *J. Appl. Phys.* **108**, 054504 (2010).

3. Golubev T., Liu D., Lunt R., & Duxbury P. Understanding the impact of C60 at the interface of perovskite solar cells via drift-diffusion modeling. *AIP Adv.* **9**, 035026 (2019).

4. Kern J., Schwab S., Deibel C., & Dyakonov V. Binding energy of singlet excitons and charge transfer complexes in MDMO-PPV:PCBM solar cells. *Phys. Status Solidi RRL* **5**, 364-366 (2011).

5. Rubel O., Baranovskii S. D., Stolz W., & Gebhard F. Exact solution for hopping dissociation of geminate electron-hole pairs in a disordered chain. *Phys. Rev. Lett.* **100**, 196602 (2008).

6. Schlaf R., Murata H., & Kafafi Z. H. Work function measurements on indium tin oxide films. *J. Electron. Spectrosc. Relat. Phenom.* **120**, 149-154 (2001).

7. Kyaw A. K. K., Sun X. W., Jiang C. Y., Lo G. Q., Zhao D. W., & Kwong D. L. An inverted organic solar cell employing a sol-gel derived ZnO electron selective layer and thermal evaporated MoO3 hole selective layer. *Appl. Phys. Lett.* **93**, 221107 (2008).

8. Zhou H., *et al.* Polymer homo-tandem solar cells with best efficiency of 11.3%. *Adv. Mater.* **27**, 1767-1773 (2015).

9. Wei Q., Mukaida M., Naitoh Y., & Ishida T. Morphological change and mobility enhancement in PEDOT:PSS by adding co-solvents. *Adv. Mater.* **25**, 2831-2836 (2013).

10. Torabi S., *et al.* Strategy for enhancing the dielectric constant of organic semiconductors without sacrificing charge carrier mobility and solubility. *Adv. Funct. Mater.* **25**, 150-157 (2015).

11. Tao Y., Yang C., & Qin J. Organic host materials for phosphorescent organic light-emitting diodes. *Chem. Soc. Rev.* **40**, 2943-2970 (2011).

12. Nakanotani H., *et al.* High-efficiency organic light-emitting diodes with fluorescent emitters. *Nat. Commun.* **5**, 4016 (2014).

13. Hasan M., *et al.* Exciton–exciton annihilation in thermally activated delayed fluorescence emitter. *Adv. Funct. Mater.* **30**, 2000580 (2020).
